# Health system readiness to deliver integrated early childhood interventions: a scoping review protocol

**DOI:** 10.1136/bmjopen-2026-116913

**Published:** 2026-11-06

**Authors:** Ayobami Adebayo Bakare, Faith A Ajayi, Japhet Y Galaboyi, Gbemisola Tina Olubayo, Olumide Ebenezer Olufayo, Obioma Uchendu

**Affiliations:** 1 Department of Community Medicine, University College Hospital, Ibadan, Nigeria; 2 Department of Global Public Health, Karolinska Institutet, Stockholm, Sweden; 3 Oxygen for Life Initiative, Ibadan, Nigeria; 4 Department of Community Medicine, University of Ibadan, Ibadan, Nigeria

**Keywords:** Health Services, PUBLIC HEALTH, Community child health

## Abstract

**Abstract:**

**Introduction:**

Early childhood represents a critical window for shaping the trajectory of an individual’s health, learning and overall well-being. Global frameworks such as the WHO Nurturing Care Framework emphasise the health system as a crucial platform for scaling up integrated early childhood development (ECD) interventions. Yet, significant gaps persist regarding how prepared health systems are to implement these interventions sustainably and at scale, which readiness strategies are commonly applied, and which components remain neglected. This scoping review aims to synthesise global evidence on health system readiness to deliver integrated ECD interventions. Specifically, the review seeks to: (1) map existing health system readiness strategies for integrated ECD delivery, (2) identify components that are commonly implemented versus those that are underused or absent, and (3) highlight gaps and opportunities for strengthening health systems to support holistic ECD at scale.

**Methods and analysis:**

The review will follow the methodological framework of Arksey and O’Malley (2005) and the Joanna Briggs Institute Manual for Evidence Synthesis (2020). Searches will be conducted across PubMed/MEDLINE, Scopus, Web of Science, CINAHL, PsycINFO, Google Scholar and relevant grey literature, restricted to sources published from 2000 to 2026. Title and abstract screening will be performed independently by two reviewers using Covidence/Rayyan, with full-text screening conducted in duplicate. The study selection process will be documented using a Preferred Reporting Items for Systematic Reviews and Meta-Analyses (PRISMA-ScR) flow diagram. Data extraction will be drafted in Excel, and any discrepancies will be discussed and reconciled by a senior reviewer; also, no critical appraisal will be undertaken as the aim is to map interventions rather than to assess effectiveness.

**Ethics and dissemination:**

This review has been registered with the Open Science Framework (https://doi.org/10.17605/OSF.IO/U7PTK). Ethical approval has been obtained from the University of Ibadan/University College Hospital Ethics Committee (UI/EC/01210). Findings will be disseminated through submission to a peer-reviewed journal.

**STRENGTHS AND LIMITATIONS OF THE STUDY**

- The review will be conducted using a robust methodological approach based on Joanna Briggs Institute guidance, ensuring rigour and transparency.
- A comprehensive search strategy across multiple databases and grey literature sources will maximise the breadth and inclusiveness of the evidence base.
- Restricting the review to English language publications may introduce language bias and exclude relevant evidence.
- Heterogeneity in definitions of ‘readiness’ and inconsistencies in reporting across published studies and grey literature may affect comparability and synthesis.
- The broad scope may reduce depth of analysis and, as a scoping review, intervention effectiveness or methodological quality will not be formally assessed.

## Introduction

 Early childhood represents a critical window for shaping lifelong health, learning and well-being.^1^ During these formative years, children require nurturing care, including good health, adequate nutrition, protection, responsive caregiving and early learning opportunities to reach their full developmental potential.^2^ Yet, millions of children in low-income and middle-income countries remain at risk of suboptimal development due to undernutrition, inadequate stimulation, poverty and psychosocial stressors.^3 4^ Evidence consistently demonstrates that investing in the early years is among the most cost-effective strategies for reducing inequality, breaking cycles of poverty and enhancing national productivity.^2 5 6^

Health systems occupy a uniquely strategic position in advancing early childhood development (ECD) because of their routine engagement with families during pregnancy, childbirth and the first 1000 days of life. Through reproductive, maternal, newborn and child health services, including nutrition, mental health and early learning counselling, health systems directly influence developmental outcomes and provide critical opportunities to integrate nurturing care into existing service delivery platforms.^2 7 8^ Despite this potential, integration of ECD into health systems has progressed more slowly than other child survival priorities, leaving many developmental needs undetected or insufficiently addressed.^9–13^

Current evidence suggests that health systems are only partially ready to deliver integrated ECD interventions, with readiness varying across contexts and hinging on systemic, workforce and governance factors. In Bangladesh, integration into primary healthcare was feasible and effective but contingent on health workers’ workload, education, supervision and motivation, raising questions about sustainability without external support.^10^ Broader analyses emphasise that capacity building requires coordinated intersectoral efforts, adequate financing, governance, workforce development and reliable data systems, yet these elements remain inconsistently established across low-income and middle-income countries.^11 12^ In high-income settings, structural, organisational and financial constraints continue to limit system capacity to identify and respond to developmental risks.^13^ Collectively, these findings indicate that while health systems demonstrate potential through existing platforms and policies, significant gaps in workforce capacity, governance, financing and system alignment persist.

Furthermore, health systems have institutionalised health and nutrition components of the Nurturing Care Framework (NCF), while strategies for responsive caregiving, early learning and, especially, safety and security remain less systematically integrated.^14–17^ Commonly implemented approaches include community-based platforms, facility-based counselling, blended service delivery and technology-enabled supports, often leveraging frontline health workers and community networks, as seen in India’s public health system.^18^ Global scaling efforts, such as Reach Up and Care for Child Development, highlight intersectoral collaboration, government ownership, workforce training and monitoring systems as key strategies, though financial planning and cost documentation are frequently omitted.^19^ WHO guidelines further recommend embedding psychosocial interventions for maternal mental health and combining nutrition with responsive caregiving and early learning, yet they stop short of detailing specific interventions.^2^ Thus, while progress has been made in integrating health and nutrition, gaps persist in safety/security, referral linkages, early identification of developmental delays and financial strategies, limiting comprehensive readiness for integrated ECD delivery within health system.

Systemic challenges further constrain readiness, intersectoral collaboration remains fragile or absent, limiting the ability to mobilise actors and resources across health, education and social protection sectors.^20^ Weak data systems undermine effective implementation, with incomplete records and untimely reporting constraining monitoring and decision-making. However, rapid-cycle evaluation and learning approaches offer opportunities to strengthen coverage and quality in resource-limited settings.^21^ At the global level, many young children still lack access to quality ECD services due to poor systems-level coordination and unsystematic scaling, as efforts have largely focused on service delivery rather than institutional and governance structures.^5^

Overall, the literature underscores that while service-level innovations exist, readiness is undermined by gaps in intersectoral collaboration, data quality and systems-level governance; thus opportunities for improvement lie in strengthening collaborative frameworks, embedding continuous learning mechanisms and adopting systems approaches that align NCF for ECD interventions with broader health and social service infrastructures.^11 20 21^ This review will provide the first comprehensive global synthesis of health system readiness strategies for ECD, offering actionable insights for policymakers and practitioners by mapping readiness strategies and identifying gaps. By synthesising global evidence, it will inform priority setting and resource allocation for health system strengthening, guiding efforts to embed holistic ECD interventions into routine service delivery at scale.

For the purposes of this review, ‘health system readiness’ is defined as the ability of health systems to adopt, implement and sustain integrated ECD interventions. It encompasses workforce knowledge and skills, organisational capacity, governance and financing, service delivery arrangements and implementation preparedness. ‘Integrated ECD interventions’ are defined as strategies that embed one or more domains of the NCF (health, nutrition, responsive caregiving, early learning and safety/security) into existing maternal, newborn and child health services or that link health systems with education and social protection sectors. ‘Readiness strategies’ refer to actions undertaken to strengthen health system building blocks—including service delivery, workforce, financing, governance and information systems—in order to support the effective integration of ECD interventions.

## Methods and analysis

This scoping review will be conducted following the methodological framework proposed by Arksey and O’Malley,^22^ which outlines five key stages: identifying the research question, identifying relevant studies, study selection, charting the data and collating, summarising and reporting the results. In addition, guidance from the updated Joanna Briggs Institute (JBI) Manual for Evidence Synthesis will be applied, which expands the framework to include a sixth step ‘consultation with stakeholders’ to ensure systematic and transparent conduct of the review.^23^ Stakeholder engagement will involve consultation with members of an established international special interest group dedicated to volunteers and the third sector in the design and delivery of integrated care. Input will be sought through structured workshops and targeted discussions. Contributions will be recorded to validate findings prior to interpretation, identify potential gaps in the scoping exercise and enhance the relevance of the review to policy and practice. Stakeholder perspectives will be integrated into the synthesis by expanding the interpretation of findings and their contributions will be acknowledged in the discussion to illustrate how they shaped the final report.

In addition, this scoping reviews will systematically evaluate health system readiness for the implementation of integrated ECD interventions. These reviews will be complemented by a global synthesis of data to identify strengths, gaps and opportunities across diverse contexts. Input from international experts and networks will refine the reviews and inform subsequent research proposals. Dissemination activities will include peer-reviewed publications, presentations at local, national and international conferences, and targeted outputs such as policy briefs designed to inform decision makers and practitioners. This comprehensive dissemination strategy will ensure that findings are rigorously communicated, widely accessible and positioned to influence both policy and practice at multiple levels.

These guidelines support clear documentation of the objectives, inclusion criteria, search strategy and analytic approach prior to initiating the review. Furthermore, this protocol adheres to the Preferred Reporting Items for Systematic Reviews and Meta-Analyses extension for Scoping Reviews (PRISMA-ScR; online supplemental material 1),^24^ which provides a standardised framework to enhance reporting quality, transparency and reproducibility.

### Determining research questions

Members of this research team have previously worked to identify high-priority research questions aimed at strengthening health system capacity for the delivery of integrated ECD interventions across diverse global contexts. During a consensus meeting, several critical issues were raised regarding the role of health systems and their readiness to implement ECD programmes, which directly informed the formulation of the research question for this scoping review. In addition, team feedback emphasised that the inquiry should not be restricted to a single child population, as programmes developed for other vulnerable groups may provide transferable insights and lessons for ECD delivery.

Accordingly, this review seeks to address the following overarching research questions:

1. How ready are health systems to deliver integrated ECD interventions?
2. Which strategies for delivering integrated ECD interventions are commonly implemented within health systems and which strategies are omitted?
3. What gaps exist in the delivery of integrated ECD interventions and what opportunities are there for improvement within the health system?

The review has the following specific objectives:

1. Map out health system readiness to deliver integrated ECD interventions.
2. Examine which strategies are commonly implemented.
3. Identify current gaps in integrated ECD delivery and opportunities for improvement.

### Search strategy

The search strategy will be developed collaboratively by the review team to ensure comprehensive and reproducible identification of relevant literature on health system readiness to deliver integrated ECD interventions. The strategy will be informed by the NCF domains of responsive caregiving, opportunities for early learning and safety and security in global contexts, these domains remain understudied.^25^

A systematic search will be conducted across the following databases: PubMed, Scopus, Web of Science, CINAHL and PsycINFO. Grey literature sources will include reports from UNICEF, WHO, World Bank, national ministries of health, non-governmental organisation publications and conference proceedings related to ECD and health system readiness. Only grey literature containing empirical findings (such as evaluations, surveys or implementation studies) will be included to ensure that the evidence base is grounded in data rather than solely policy or opinion pieces. Google Scholar will be searched systematically, screening the first 200 results per query using predefined keywords related to ECD and integrated health system interventions. Backward citation tracking (reference lists) and forward citation tracking (studies citing included articles) will be conducted to ensure comprehensive coverage. The date will be restricted to 2000 and 2026 because integrated ECD interventions and health system readiness gained prominence after 2000, making earlier studies less relevant. Extending the search period ensured adequate coverage across domains that remain relatively underexplored. Only studies conducted in global contexts will be included and eligibility will be limited to publications with full-text access in English due to lack of translation capacity and resource constraints. At the time of submitting this protocol, the search has not yet been conducted; the search will be carried out in November 2026 and completed in December 2026.

The search strategy will combine controlled vocabulary (eg, MeSH terms) with free-text keywords related to health system readiness, integrated ECD interventions and global implementation. All search results will be imported into Zotero for reference management. Duplicate records will be removed both automatically and manually before screening. Screening will be conducted in Covidence, encompassing both title/abstract and full-text review stages. An example of the complete search strategy for PubMed is provided in table 1 and online supplemental file and will be adapted appropriately for other databases.

**Table 1:** A sample of database search strategy

Database	Search strategy
PubMed	(("Delivery of health care"[MeSH Terms] OR "health system*"[All Fields] OR "health facilit*"[All Fields] OR "health* worker*"[All Fields]) AND ("Early childhood development"[All Fields] OR "Early childhood stimulation"[All Fields] OR "responsive caregiving"[All Fields] OR "parent* stimulation"[All Fields] OR "home interaction*"[All Fields] OR ("safety"[MeSH Terms] OR "safety"[All Fields]) OR "security"[All Fields]) OR "opportunit* for learning*"[All Fields]) AND ("availab*"[All Fields] OR "read*"[All Fields] OR "willing*"[All Fields]) AND ("methods"[MeSH Terms] OR "intervention*"[All Fields] OR "deliver*"[All Fields])) AND (2000:2026[pdat])

### Inclusion criteria

In this review, included interventions will be mapped to the NCF domains (health, nutrition, responsive caregiving, early learning and safety/security), while readiness factors will be mapped to the WHO health system building blocks (service delivery, workforce, financing, governance and information systems). This dual framework will guide both study selection and analysis, ensuring that interventions and readiness are systematically distinguished and assessed.^2 26^ Therefore, the authors will adopt the PCC (population, concept and context) framework which is specifically recommended by the JBI Manual for Evidence Synthesis for scoping reviews.^27^

#### Population

Health systems (including healthcare workers, managers, policymakers) involved in delivering ECD interventions.

#### Concept

Health system readiness (capacity, resources, governance, workforce, information systems, financing, service delivery) to deliver integrated ECD interventions.

#### Context

Global health system settings (any country or region; primary care, community health, facility-based settings).

### Types of evidence to be included

All records retrieved from the database and grey literature searches will be screened against the predefined eligibility criteria. The screening process will be conducted in two stages: (1) title and abstract screening and (2) full-text screening.

Title and abstract screening will be conducted independently by two reviewers using the review management software Covidence.^28^ Each record will be evaluated to determine whether it meets the core eligibility criteria, including studies examining health system readiness in the delivery of integrated childhood interventions; a focus on at least one NCF domain (responsive caregiving, opportunities for early learning, security and safety); global scope; and eligible study designs (quantitative, qualitative, mixed-methods, health policy and systems research, government or agency policy/strategy documents and technical reports). Existing reviews (eg, scoping review, narrative review, systematic review etc.) will not be included but will be used only to identify additional primary studies.

Full-text screening will also be conducted in duplicate by the same two independent reviewers using Covidence. Discrepancies during full-text screening will be resolved through team discussion, and if unresolved, adjudicated by a senior reviewer (AAB).

### Data extraction/charting

The entire study selection process will be using a PRISMA-ScR flow diagram, including the number of records retrieved, the number remaining after duplicate removal, the number screened at the title and abstract level, the number assessed at the full-text level and the number included in the final synthesis. This rigorous multimodal screening approach ensures transparency, reproducibility and adherence to methodological standards consistent with PRISMA-ScR guidance.

Data extraction will initially be drafted in Excel. For each included study, the following information will be systematically charted: author (year, country, setting); study design and methods; population/level of the health system; description of integrated ECD interventions (components, delivery platform); health system readiness domains reported (capacity, human resources for health, health information systems, financing, governance, service delivery, tools/resources); strategies implemented to strengthen readiness and the domains they target; and reported gaps, barriers and opportunities for improvement (table 2).

**Table 2:** Data extraction plan

Category	Data to be extracted
Article information	Author(s), year of publication, country, region, funding source
Study characteristics	Study design, study setting (urban/rural/peri-urban), population characteristics (age group, target beneficiaries), level of healthcare system (primary, secondary, tertiary)
Intervention details	Description of integrated ECD interventions (components, delivery platform, duration, intensity), stakeholders engaged (government, NGOs, community health workers, parents/caregivers), theoretical/conceptual framework used
Health system readiness	Domains assessed (capacity, human resources for health, health information systems, financing, governance, service delivery, tools/resources), alignment with WHO health system building blocks
Strategies implemented	Strategies to strengthen readiness, domains targeted, mechanisms of implementation, integration approaches
Outcomes reported	Key findings related to readiness, gaps identified, opportunities for improvement, lessons learnt
Other contextual factors	Barriers and facilitators, sociocultural context, sustainability considerations, equity dimensions (gender, socioeconomic status)

ECD, early childhood development; NGO, non-governmental organisation.

Two reviewers (JYG and FAA) will independently extract data from each included study. Following independent extraction, harmonisation will be performed and discrepancies will be resolved through discussion between both reviewers. In cases where consensus cannot be reached, adjudication will be undertaken by a senior author (OU). Where multiple publications originate from the same study, they will be treated as a single unit of evidence. Data will be extracted across all linked publications and integrated into one record within the charting matrix. A log of linked ‘friend studies’ will be maintained to ensure transparency, avoid double-counting, and track relationships between papers.

#### Critical appraisal of available literature

In line with the primary aim of this scoping review, which is to provide an overview of the included studies, we will not conduct a formal risk of bias assessment for individual studies, and no study will be excluded based on quality assessment.

##### Analysis and presentation of results

All collected information will be checked for accuracy, consistency and completeness. Data cleaning will be conducted in Microsoft Excel, with support from Covidence for screening records. Any inconsistencies or missing values will be cross-checked against the original articles, and discrepancies will be resolved collaboratively by the review team.

A PRISMA-ScR flow diagram will be used to illustrate the number of studies identified, screened, assessed for eligibility and included in the final synthesis.

Data will be synthesised both narratively and descriptively, in alignment with the objectives of the scoping review. Quantitative findings will be summarised using descriptive statistics where appropriate. Qualitative findings will be organised thematically. Included interventions will be mapped to the NCF domains, while readiness factors will be mapped to the WHO health system building blocks. Barriers and facilitators will be coded thematically to enable cross-study comparison, and strategies will be categorised as commonly implemented, underused or omitted based on frequency of reporting across included studies. This structured synthesis will allow identification of readiness strategies, underused components, gaps and opportunities for strengthening integrated ECD within health systems.

Results will be presented using: tables summarising characteristics of included studies, matrices mapping intervention types to NCF domains, charts/figures illustrating regional distribution and intervention focus, and narrative summaries interpreting trends, gaps and implementation strategies.

## Patient and public involvement

Patients or the public were not involved in the design, conduct, reporting or dissemination of our research.

## Ethics and dissemination

This review relies exclusively on publicly available published data and does not involve direct data collection from human participants. Nevertheless, ethical approval was sought and obtained from the University of Ibadan/University College Hospital Ethics Committee (UI/EC/01210) in line with institutional requirements and local committee recommendations. The rationale for securing ethics approval was to ensure compliance with governance standards, provide independent oversight of the protocol and enhance transparency and credibility of the review process. The protocol has been registered with the Open Science Framework (https://doi.org/10.17605/OSF.IO/U7PTK). The study team will oversee all stages of the review, with roles of external collaborators or stakeholders documented where applicable. Findings will be disseminated through peer-reviewed publications, conference presentations and targeted outputs such as policy briefs to inform decision makers and practitioners. This dissemination strategy is designed to maximise visibility and impact, contributing to global efforts to strengthen health system readiness for integrated childhood interventions and to promote balanced investments across the domains of the NCF.

## Discussion

### Anticipated contribution

This scoping review will provide the first comprehensive global synthesis of health system readiness for integrated ECD delivery. By systematically mapping strategies, identifying gaps and highlighting opportunities, the review will inform policymakers, practitioners and researchers about priority areas for health system strengthening.

### Potential limitations

Several limitations should be acknowledged. First, restricting the review to English-language publications may introduce language bias and exclude relevant evidence published in other languages. Second, excluding studies published before 2000 may overlook foundational work on health system readiness for ECD. Third, because we will not conduct a formal quality appraisal, the review may include studies with varying methodological rigour. Finally, the broad scope of the review covering global evidence and multiple study designs may result in heterogeneous findings that are challenging to synthesise cohesively.

### Implications for policy and practice

Findings from this review are expected to provide actionable insights for health system decision-makers. By identifying which readiness strategies are commonly implemented and which are neglected, the review will support targeted investments in health system building blocks, including workforce training, health information systems, governance mechanisms and financing for integrated ECD.

### Implications for research

This review will highlight evidence gaps and methodological limitations in the current literature, informing the design of future primary research on health system readiness for ECD. It may reveal the need for standardised measurement tools, rigorous implementation research and cost-effectiveness analyses.

### Anticipated challenges

Synthesising evidence across diverse study designs (quantitative, qualitative and mixed methods), multiple countries with varying health system contexts and heterogeneous definitions of ‘readiness’ may pose challenges. The review team will address these through careful data charting, narrative synthesis and transparent reporting of variability.

### Data availability statement

This study is a scoping review protocol. No primary data were generated or collected. All data will be obtained from published articles and grey literature identified through the search strategy. The data extraction forms and charted results will be made available on reasonable request to the corresponding author.

## Supplementary material

10.1136/bmjopen-2026-116913 online supplemental file 1

10.1136/bmjopen-2026-116913 online supplemental file 2
