# Supplementary material for: Health system readiness to deliver integrated early childhood interventions: a scoping review protocol
Source: BMJ Open. 2026 Nov 6;16(11):e116913. doi: 10.1136/bmjopen-2026-116913 (PMC13756350; doi:10.1136/bmjopen-2026-116913)
Supplement: Supplementary file 1 [file bmjopen-16-11-s001.pdf]

Table 1: Full Database search strategy

Database	Search Strategy
**Pubmed**	(("Delivery of health care"[MeSH Terms] OR "health system*"[All Fields] OR "health facilit*"[All Fields] OR "health* worker*"[All Fields]) AND ("Early childhood development"[All Fields] OR "Early childhood stimulation"[All Fields] OR "responsive caregiving"[All Fields] OR "parent* stimulation"[All Fields] OR "home interaction*"[All Fields] OR ("safety"[MeSH Terms] OR "safety"[All Fields]) OR "security"[All Fields]) OR "opportunit* for learning*"[All Fields]) AND ("availab*"[All Fields] OR "read*"[All Fields] OR "willing*"[All Fields]) AND ("methods"[MeSH Terms] OR "intervention*"[All Fields] OR "deliver*"[All Fields])) AND (2000:2026[pdat])
**Web of science**	"Delivery of health care" OR "Health System*" OR "Health facilit*" OR "Health* worker*" (Topic) and "Early childhood development" OR "Early childhood stimulation" OR "responsive caregiving" OR "Parent* stimulation" OR "Home interaction*" OR safety OR security OR "opportunit* for learning*" (Topic) and Availab* OR Read* OR Willing* (Topic) and "methods" OR Intervention* OR Deliver* (All Fields)
**CINAHL**	("Delivery of health care" OR "Health System*" OR "Health facilit*" OR "Health* worker*") AND ("Early childhood development" OR "Early childhood stimulation" OR "responsive caregiving" OR "Parent* stimulation" OR "Home interaction*" OR safety OR security OR "opportunit* for learning*") AND (Availab* OR Read* OR Willing*) AND ("methods" OR Intervention* OR Deliver*)
**PsycInfo**	(("Delivery of health care" OR "Health System*" OR "Health facilit*" OR "Health* worker*") AND (("Early childhood development" OR "Early childhood stimulation" OR "responsive caregiving" OR "Parent* stimulation" OR "Home interaction*" OR safety OR security OR "opportunit* for learning*") AND (Availab* OR Read* OR Willing*) AND (("methods" OR Intervention* OR Deliver*)
**SCOPUS**	(TITLE-ABS-KEY ("Health system" OR "Health facilit" OR "Health worker*") AND TITLE-ABS-KEY ("Early childhood development" OR "Early childhood stimualtion" OR "responsive caregiving" OR "parent* stimulation" OR "Home interaction" OR safety OR security OR "opportunit* for learning*") AND TITLE-ABS-KEY (availab* OR read* OR willing*) AND TITLE-ABS-KEY (intervention* OR deliver*))

Filters applied:

- Study period limited to publications from 2000 to 2026.
- conference papers, preprints, commentaries, abstracts, opinion pieces lacking empirical findings, and book chapters without empirical findings.
- Studies not involving human subjects, and not published in English.
