# Supplementary material for: Health system readiness to deliver integrated early childhood interventions: a scoping review protocol
Source: BMJ Open. 2026 Nov 6;16(11):e116913. doi: 10.1136/bmjopen-2026-116913 (PMC13756350; doi:10.1136/bmjopen-2026-116913)
Supplement: Supplementary file 2 [file bmjopen-2026-116913.reviewer_comments.pdf]

PEER REVIEW HISTORY

BMJ Open publishes all reviews undertaken for accepted manuscripts. Reviewers are asked to complete a checklist review form and are provided with free text boxes to elaborate on their assessment. These free text comments are reproduced below.

ARTICLE DETAILS

Title (Provisional)

Health System Readiness to Deliver Integrated Early Childhood Interventions: A Scoping Review Protocol

Authors

Bakare, Ayobami Adebayo; Ajayi, Faith A; Galaboyi, Japhet Y; Olubayo, Gbemisola T; Olufayo, Olumide E; Uchendu, Obioma

---

VERSION 1 - REVIEW

**Reviewer**	**1**
**Name**	**Osborne, Augustus**
**Affiliation**	**Njala University, Department of Biological Sciences**
**Date**	**04-Apr-2026**
**COI**	**None**

Thank you for the opportunity to review this protocol. The manuscript addresses a worthwhile and timely topic. A scoping review on health system readiness to deliver integrated early childhood development interventions is well suited to the current literature, where evidence is spread across implementation studies, programme reports, policy documents, and health systems research. The topic is appropriate for BMJ Open, and the manuscript has several strengths, including protocol registration, use of established scoping review guidance, duplicate screening, and inclusion of grey literature.

That said, the protocol requires major revision before it is ready for publication. The main issue is not the choice of topic or study design, but the lack of clarity and consistency in the way the review is conceptualised and operationalised. At present, the review question is broader than the methods can support, and several core terms remain insufficiently defined.

My main comments are as follows.

First, the central concepts need much sharper definition. The manuscript refers repeatedly to health system readiness, integrated ECD interventions, readiness strategies, health system building blocks, and Nurturing Care Framework domains, but these are not clearly distinguished from one another. In particular, “health system readiness” needs an explicit

operational definition. It is not clear whether readiness refers mainly to workforce knowledge and skills, organisational capacity, governance and financing, service delivery arrangements, implementation preparedness, or all of these. Likewise, "integrated ECD interventions" needs to be defined more precisely. The manuscript should make clear what kind of integration qualifies a study for inclusion, for example integration into maternal and child health services, integration across Nurturing Care domains, or integration across sectors.

Second, the eligibility criteria do not align well with the stated review questions. The review questions are broad and system-level, but the Population element in the inclusion criteria focuses on healthcare workers' knowledge, skills, perceptions, beliefs, and attitudes. This is much narrower than the stated aim of assessing health system readiness. Many relevant studies may focus on governance, supervision, financing, infrastructure, service platforms, or programme design without examining provider attitudes directly. I suggest revising the eligibility framework so that it is more clearly built around concept and context rather than a narrow population formulation.

Third, the manuscript needs to explain much more clearly how the WHO health system building blocks and the Nurturing Care Framework will be used together. At present, both frameworks are mentioned, but the relationship between them is not specified. These frameworks describe different things: one concerns the functions of the health system, the other concerns domains of child and caregiver support. The protocol would be much stronger if the authors clearly stated that included interventions will be mapped to Nurturing Care domains, while readiness factors will be mapped to health system building blocks, or another similarly explicit analytic framework.

Fourth, the search strategy is not yet developed enough for a protocol paper. The manuscript names the databases and gives broad keyword groupings, but it does not provide a full reproducible search strategy for at least one database. Some of the terms listed are extremely broad and are likely to retrieve a large volume of irrelevant material. The grey literature approach is also too vague. The authors should specify exactly which grey literature sources will be searched, how Google Scholar will be used, whether citation tracking will be conducted, and why the date restriction from 2000 to 2025 was chosen. The English-language restriction should also be justified and acknowledged as a limitation.

Fifth, there is an internal contradiction regarding critical appraisal. The abstract states that no critical appraisal will be undertaken, but the methods section later states that a quality appraisal will be conducted using the CASP tools. These positions are not consistent. The authors need to decide whether they will conduct critical appraisal and then describe that approach consistently throughout the manuscript. If no appraisal will be conducted, that is acceptable for a scoping review, but the manuscript should say so clearly. If appraisal will be conducted, the rationale, tools, and intended use of those findings should be stated explicitly.

Sixth, the planned synthesis remains too general. The manuscript says that data will be synthesised narratively and descriptively, but the protocol does not explain clearly how the review will move from extracted data to conclusions about readiness strategies, underused components, gaps, and opportunities. The manuscript would benefit from a more explicit synthesis plan. For example, the authors could specify whether they will use a deductive matrix based on the WHO building blocks and Nurturing Care domains, whether thematic coding will be used for barriers and facilitators, and how they will identify strategies that are "commonly implemented" or "omitted."

Seventh, the protocol should clarify how it will handle existing reviews. At present, the authors appear willing to include narrative reviews, scoping reviews, and systematic reviews among eligible evidence sources. That may be possible, but it raises the risk of double counting evidence if primary studies are also included. The protocol should clarify whether reviews will be included as evidence sources in their own right, or used only to identify additional primary studies.

Eighth, the ethics section should be clarified. This review uses publicly available published data and does not involve direct human participation. In most cases, formal ethics approval is not required for such work. The manuscript states that ethics approval has been obtained, which is not necessarily problematic, but the authors should explain why approval was sought and whether it relates specifically to the scoping review or to a wider programme of work.

Ninth, the strengths and limitations section should be revised. At present it is too generic and does not fully reflect the likely methodological limitations of the planned review. More useful limitations would include heterogeneity in definitions of readiness, likely inconsistency in reporting across studies and grey literature, language restriction, and the inability of a scoping review to assess intervention effectiveness.

Finally, the manuscript would benefit from a thorough language and style edit. The English is generally understandable, but there are several awkward sentences, minor grammatical problems, inconsistent terminology, and places where the wording is imprecise. The references also need attention, as some are incomplete or not formatted consistently.

**Reviewer**	**2**
**Name**	**Amir-Behghadami, Mehrdad**
**Affiliation**	**Tabriz University of Medical Sciences, Health Service Management**
**Date**	**29-Jun-2026**
**COI**	**None**

GENERAL ASSESSMENT

This protocol outlines a scoping review that aims to synthesize global evidence on health system readiness to deliver integrated early childhood development (ECD) interventions. The topic is timely and relevant given the global emphasis on the WHO Nurturing Care Framework and the need to strengthen health systems for integrated ECD delivery. However, several methodological and structural issues require attention before the protocol can be accepted for publication.

MAJOR CONCERNS

1. Title

Issue: The current title does not identify the manuscript as a study protocol, which is a requirement for BMJ Open protocols.

Recommendation: The authors should revise the title to clearly indicate that this is a protocol for a scoping review.

Suggested Title Options:

• "Health System Readiness to Deliver Integrated Early Childhood Interventions: A Scoping Review Protocol"

• "Scoping Review Protocol: Health System Readiness for Integrated Early Childhood Development Interventions"

• "Health System Readiness to Deliver Integrated Early Childhood Interventions: Protocol for a Scoping Review"

2. Abstract

Issue: While the abstract provides a general overview, the study purpose could be stated more explicitly and with greater clarity.

Recommendation: Revise the abstract to include a clearer, more concise statement of the specific study objectives. The current abstract mentions mapping existing strategies and identifying gaps but could be more precise about what the review will specifically achieve.

Suggested Revision for Abstract (Introduction section):

"This scoping review aims to synthesize global evidence on health system readiness to deliver integrated early childhood development interventions. Specifically, the review seeks to: (1) map existing health system readiness strategies for integrated ECD delivery, (2) identify components that are commonly implemented versus those that are underutilized or absent, and (3) highlight gaps and opportunities for strengthening health systems to support holistic ECD at scale."

3. Introduction

Issue: The introduction lacks a clear "part-to-whole" structure and does not adequately situate the study within the broader field. Additionally, the contribution of the study and its

specific contribution to the specialty/field is not clearly articulated at the end of the introduction.

Recommendation: Restructure the introduction following a logical progression:

1. Part (broad context): Global burden of ECD challenges and importance of early childhood

2. Part (narrowing): Current limitations in health system integration of ECD, fragmented service delivery

3. Part (specific gap): Evidence gaps regarding health system readiness strategies, implementation challenges, and what components are neglected

4. Whole (study contribution): Clearly state how this scoping review will address these gaps and contribute to the field

Additional Content Needed:

The introduction should explicitly state at the end:

• The contribution of this study to the field (e.g., "This review will provide the first comprehensive global synthesis of health system readiness strategies for ECD, offering actionable insights for policymakers and practitioners")

• How this study advances the specialty (e.g., "By mapping readiness strategies and identifying gaps, this review will inform priority setting and resource allocation for health system strengthening")

4. Methods - Arksey and O'Malley Framework

Issue: The authors reference Arksey and O'Malley's (2005) five-step framework but fail to acknowledge that updated guidance has expanded this to six steps. The newer JBI Manual for Evidence Synthesis (2020) and the updated methodological guidance by Peters et al. (2020) outline six stages.

Critical Problem: The current manuscript does not address Step 6: "consultation with stakeholders," which is a crucial component of the updated framework. The authors mention "knowledge translation" and engaging with a "special interest group," but this is not explicitly framed as the sixth step of the methodological framework.

Recommendation: The methods section should be restructured to follow the six-step framework:

1. Identifying the research question

2. Identifying relevant studies

3. Study selection

4. Charting the data

5. Collating, summarizing, and reporting the results

6. Consultation with stakeholders (NEW STEP)

Required Revision:

The authors should explicitly include Step 6 in their methodology and describe:

• Who will be consulted (the "international special interest group" mentioned in the knowledge translation section should be integrated here)

• How consultation will be conducted (e.g., workshops, interviews, surveys)

• The purpose of consultation (e.g., to validate findings, enhance relevance, inform interpretation)

• How stakeholder input will be integrated into the review findings

5. PRISMA Guidelines

Issue: The manuscript incorrectly states that the review will follow PRISMA-ScR (which is correct), but there is inconsistency and potential confusion in the text regarding PRISMA guidelines.

Specific Issues:

• The manuscript correctly references PRISMA-ScR (Tricco et al., 2018) in multiple places

• However, the authors state: "The study selection process will be documented using a PRISMA flow diagram" - this should specifically be a PRISMA-ScR flow diagram

• The authors have completed the PRISMA-ScR checklist (appears as supplementary material), which is appropriate

Recommendation:

• Consistently use "PRISMA-ScR" terminology throughout the manuscript rather than generic "PRISMA"

• Ensure the PRISMA-ScR checklist is properly completed with page numbers for each item (the current checklist appears to have missing page numbers in the "REPORTED ON PAGE #" column)

• Reference the PRISMA-ScR paper correctly and consistently

6. Eligibility Criteria Framework

Issue: The authors use a Population-Intervention-Outcome-Study design (PIOS) framework without providing a citation or justification. More critically, PIOS is not the most appropriate framework for scoping reviews.

Recommendation: The authors should adopt the PCC (Population, Concept, Context) framework, which is specifically recommended by the JBI Manual for Evidence Synthesis for scoping reviews, OR the PICOS framework for systematic reviews.

Suggested Revision Using PCC:

PCC Element Description for This Review

Population Health systems (including healthcare workers, managers, policymakers) involved in delivering ECD interventions

Concept Health system readiness (capacity, resources, governance, workforce, information systems, financing, service delivery) to deliver integrated ECD interventions

Context Global health system settings (any country or region; primary care, community health, facility-based settings)

Citation to Support PCC Framework:

Peters MDJ, Godfrey C, McInerney P, Munn Z, Tricco AC, Khalil H. Chapter 11: Scoping Reviews. In: Aromataris E, Munn Z, editors. JBI Manual for Evidence Synthesis. JBI, 2020. Available from: https://synthesismanual.jbi.global

Alternative Option (if authors prefer PICOS):

The authors could use PICOS and cite:

Amir-Behghadami M, Janati A. Population, Intervention, Comparison, Outcomes and Study (PICOS) design as a framework to formulate eligibility criteria in systematic reviews. Emerg Med J. 2020;37(6):387-387. doi:10.1136/emermed-2020-209567

7. Discussion Section

Issue: The manuscript lacks a dedicated Discussion section, which is an expected component of a protocol paper.

Recommendation: Add a Discussion section that addresses:

1. Anticipated significance and contribution - What will this review add to the existing body of knowledge?

2. Potential limitations beyond those listed in the "Strengths and Limitations" section:

o Restriction to English language publications (may introduce language bias)

o Exclusion of studies published before 2000 (may miss foundational work)

o No formal quality appraisal (may include studies with methodological limitations)

o Potential for publication bias

3. Implications for policy and practice - How will findings inform health system strengthening?

4. Implications for future research - What research gaps might be identified through this review?

5. Anticipated challenges - Challenges in synthesizing diverse evidence (quantitative, qualitative, mixed methods; peer-reviewed vs. grey literature)

Suggested Structure for Discussion Section:

text

DISCUSSION

Anticipated Contribution

This scoping review will provide the first comprehensive global synthesis of health system readiness for integrated ECD delivery. By systematically mapping strategies, identifying gaps, and highlighting opportunities, the review will inform policymakers, practitioners, and researchers about priority areas for health system strengthening.

Potential Limitations

Several limitations should be acknowledged. First, restricting to English-language publications may introduce language bias and potentially exclude relevant evidence published in other languages. Second, the exclusion of studies published before 2000 may miss foundational work on health system readiness for ECD. Third, as we will not conduct formal quality appraisal, the review may include studies with varying methodological rigor. Fourth, the broad scope of the review (global, multiple study designs) may result in heterogeneous evidence that is challenging to synthesize cohesively.

Implications for Policy and Practice

Findings from this review are expected to provide actionable insights for health system decision-makers. By identifying which readiness strategies are commonly implemented and which are neglected, the review will support targeted investments in health system building blocks, including workforce training, health information systems, governance mechanisms, and financing for integrated ECD.

Implications for Research

This review will identify evidence gaps and methodological limitations in the current literature, informing the design of future primary research on health system readiness for ECD. It may reveal the need for standardized measurement tools, rigorous implementation research, and cost-effectiveness analyses.

Anticipated Challenges

Synthesizing evidence across diverse study designs (quantitative, qualitative, mixed methods), multiple countries with varying health system contexts, and heterogeneous definitions of "readiness" may pose challenges. The review team will address these through careful data charting, narrative synthesis, and transparent reporting of variability.

MINOR CONCERNS

8. Search Strategy

Issue: The search strategy table (Table 1) is presented in a format that is somewhat difficult to read. Additionally, the authors should clearly state the date when the search will be executed.

Recommendations:

• Format Table 1 more clearly with separate rows for each concept

• State the planned search execution date

• Consider including a sample search strategy for additional databases (at least one other database) in supplementary materials

9. Data Extraction

Issue: The data extraction plan in Table 2 could be more comprehensive.

Recommendation: Add the following items to the data extraction table:

• Funding sources

• Study setting (e.g., urban, rural, national, community, facility)

• Stakeholders engaged in the intervention

• Theoretical/conceptual frameworks used

• Health system building blocks assessed (WHO framework)

10. Critical Appraisal

Issue: The authors state they will use CASP tools for quality appraisal but do not specify which specific CASP checklist(s) they will use (e.g., qualitative studies, cohort studies, etc.).

Recommendation: Specify which CASP checklists will be applied based on the study designs identified, and clarify how appraisal results will be presented (e.g., not used to exclude studies but to provide context for interpretation of findings).

11. Strengths and Limitations

Issue: The strengths and limitations section is brief and could be expanded to include methodologically relevant points.

Recommendation: Consider adding:

• Strength: Robust methodological approach based on JBI guidance

• Strength: Comprehensive search across multiple databases and grey literature

• Limitation: Potential for missing non-English publications

• Limitation: Broad scope may limit depth of synthesis for specific components

VERSION 1 - AUTHOR RESPONSE

Reviewer 1

First, the central concepts need much sharper definition. The manuscript refers repeatedly to health system readiness, integrated ECD interventions, readiness strategies, health system building blocks, and Nurturing Care Framework domains, but these are not clearly distinguished from one another. In particular, "health system readiness" needs an explicit operational definition. It is not clear whether readiness refers mainly to workforce knowledge and skills, organisational capacity, governance and financing, service delivery arrangements, implementation preparedness, or all of these. Likewise, "integrated ECD interventions" needs to be defined more precisely. The manuscript should make clear what kind of integration qualifies a study for inclusion, for example integration into maternal and child health services, integration across Nurturing Care domains, or integration across sectors.

- **Response:** we have revised the introduction to include explicit operational definitions of *health system readiness*, *integrated ECD interventions*, and *readiness strategies*. These definitions clarify scope and distinguish between readiness, building blocks, and NCF domains. The revised text now appears at the end of the introduction section. **(Page 6, line 130 – 139)**

Second, the eligibility criteria do not align well with the stated review questions. The review questions are broad and system-level, but the Population element in the inclusion criteria focuses on healthcare workers' knowledge, skills, perceptions, beliefs, and attitudes. This is much narrower than the stated aim of assessing health system readiness. Many relevant studies may focus on governance, supervision, financing, infrastructure, service platforms, or programme design without examining provider attitudes directly. I suggest revising the eligibility framework so that it is more clearly built around concept and context rather than a narrow population formulation.

- **Response:** The eligibility framework has been modified to base on PCC framework and aligned with our objective **(Page 9, Line 226 – 231)**

Third, the manuscript needs to explain much more clearly how the WHO health system building blocks and the Nurturing Care Framework will be used together. At present, both frameworks are mentioned, but the relationship between them is not specified. These frameworks describe different things: one concerns the functions of the health system, the other concerns domains of child and caregiver support. The protocol would be much stronger if the authors clearly

stated that included interventions will be mapped to Nurturing Care domains, while readiness factors will be mapped to health system building blocks, or another similarly explicit analytic framework.

- **Response:** We appreciate this observation. The *Inclusion Criteria* section has been revised to clarify that interventions will be mapped to the **Nurturing Care Framework domains**, while readiness factors will be mapped to the **WHO health system building blocks**, ensuring a clear analytic distinction between the two frameworks. **(Page 9, line 221 – 226)**

Fourth, the search strategy is not yet developed enough for a protocol paper. The manuscript names the databases and gives broad keyword groupings, but it does not provide a full reproducible search strategy for at least one database. Some of the terms listed are extremely broad and are likely to retrieve a large volume of irrelevant material. The grey literature approach is also too vague. The authors should specify exactly which grey literature sources will be searched, how Google Scholar will be used, whether citation tracking will be conducted, and why the date restriction from 2000 to 2025 was chosen. The English-language restriction should also be justified and acknowledged as a limitation.

- **Response:** Search strategy remodified and narrowed down **(page 8-9, line 218)**, description of Grey literatures names **(page 8, line 196 – 200),** google scholar **(page 8, line 200 – 202),** citation tracker **(page 8, line 202 – 204)** and the rationale for the year restriction 2000 to 2026 **(page 8, line 204 – 207)** added accordingly. English language restriction acknowledges as limitation and justified **(page 8, line 207 – 209)**

Fifth, there is an internal contradiction regarding critical appraisal. The abstract states that no critical appraisal will be undertaken, but the methods section later states that a quality appraisal will be conducted using the CASP tools. These positions are not consistent. The authors need to decide whether they will conduct critical appraisal and then describe that approach consistently throughout the manuscript. If no appraisal will be conducted, that is acceptable for a scoping review, but the manuscript should say so clearly. If appraisal will be conducted, the rationale, tools, and intended use of those findings should be stated explicitly.

- **Response:** Critical appraisal will not be conducted, in line with the primary aim of this scoping review, which is to provide an overview of the included studies. ***(page 11, line 275 – 278; Page 2 line 43)*** this is consistently stated in abstract section and the body.

Sixth, the planned synthesis remains too general. The manuscript says that data will be synthesised narratively and descriptively, but the protocol does not explain clearly how the review will move from extracted data to conclusions about readiness strategies, underused components, gaps, and opportunities. The manuscript would benefit from a more explicit synthesis plan. For example, the authors could specify whether they will use a deductive matrix based on the WHO building blocks and Nurturi]ng Care domains, whether thematic coding will be used for barriers and facilitators, and how they will identify strategies that are "commonly implemented" or "omitted."

- **Response:** The synthesis plan has been revised to clarify that quantitative findings will be summarized descriptively, qualitative findings organized thematically, interventions mapped to Nurturing Care domains, and readiness factors to WHO health system building blocks. Barriers and facilitators will be coded thematically, and strategies classified as commonly implemented, underutilized, or omitted ***(page 12, line 286 – 292)***

Seventh, the protocol should clarify how it will handle existing reviews. At present, the authors appear willing to include narrative reviews, scoping reviews, and systematic reviews among eligible evidence sources. That may be possible, but it raises the risk of double counting evidence if primary studies are also included. The protocol should clarify whether reviews will be included as evidence sources in their own right, or used only to identify additional primary studies.

- **Response:** we clarified that "existing reviews" (e.g scoping review, narrative review, systematic review etc) will not be included but will be used only to identify additional primary studies. **(page 10, line 245 – 247)**

Eighth, the ethics section should be clarified. This review uses publicly available published data and does not involve direct human participation. In most cases, formal ethics approval is not required for such work. The manuscript states that ethics approval has been obtained, which is not necessarily problematic, but the authors should explain why approval was sought and whether it relates specifically to the scoping review or to a wider programme of work.

- **Response:** Application for local ethics was done in line with recommendations from the local ethics committee **(page 12, line 305 – 308).**

Ninth, the strengths and limitations section should be revised. At present it is too generic and does not fully reflect the likely methodological limitations of the planned review. More useful limitations would include heterogeneity in definitions of readiness, likely inconsistency in reporting across studies and grey literature, language restriction, and the inability of a scoping review to assess intervention effectiveness.

- **Response:** Limitations revised, reflecting methodological limitations **(page 3, line 60 -67)**

Reviewer 2

The current title does not identify the manuscript as a study protocol, which is a requirement for BMJ Open protocols.
Recommendation: The authors should revise the title to clearly indicate that this is a protocol for a scoping review.

- **Response:** Title corrected as: *Health System Readiness to Deliver Integrated Early Childhood Interventions: A Scoping Review Protocol* ***(page 1, line 1)***

Issue: While the abstract provides a general overview, the study purpose could be stated more explicitly and with greater clarity.

Recommendation: Revise the abstract to include a clearer, more concise statement of the specific study objectives. The current abstract mentions mapping existing strategies and identifying gaps but could be more precise about what the review will specifically achieve.

- **Response:** Abstract revised to include: This scoping review aims to synthesize global evidence on health system readiness to deliver integrated early childhood development interventions. Specifically, the review seeks to: (1) map existing health system readiness strategies for integrated ECD delivery, (2) identify components that are commonly implemented versus those that are underutilized or absent, and (3) highlight gaps and opportunities for strengthening health systems to support holistic ECD at scale. ***(page 2 line 24-34)***

The introduction lacks a clear "part-to-whole" structure and does not adequately situate the study within the broader field. Additionally, the contribution of the study and its specific contribution to the specialty/field is not clearly articulated at the end of the introduction.
Recommendation: Restructure the introduction following a logical progression:
1. Part (broad context): Global burden of ECD challenges and importance of early childhood

2. Part (narrowing): Current limitations in health system integration of ECD, fragmented service delivery
3. Part (specific gap): Evidence gaps regarding health system readiness strategies, implementation challenges, and what components are neglected

- **Response** Introduction section restructured to capture:
    - Broad context: global burden of ECD challenges and the importance of early childhood. **(page 4, line 70 – 85)**
    - Narrowing: current limitations in health system integration of ECD and fragmented service delivery. **(page 4- 5, line 86 – 111)**
    - Specific gap: evidence gaps regarding health system readiness strategies, implementation challenges, and neglected components. **(page 5, line 112 – 124)**

Whole (study contribution): Clearly state how this scoping review will address these gaps and contribute to the field
Additional Content Needed:
The introduction should explicitly state at the end:
• The contribution of this study to the field

• How this study advances the specialty

- **Response:** Study contribution: clearly stating how this scoping review will address these gaps and contribute to the field. **(page 5 – 6, line 124 -129)**

Issue: The authors reference Arksey and O'Malley's (2005) five-step framework but fail to acknowledge that updated guidance has expanded this to six steps. The newer JBI Manual for Evidence Synthesis (2020) and the updated methodological guidance by Peters et al. (2020) outline six stages.
Critical Problem: The current manuscript does not address Step 6: "consultation with stakeholders," which is a crucial component of the updated framework. The authors mention "knowledge translation" and engaging with a "special interest group," but this is not explicitly framed as the sixth step of the methodological framework.

Required Revision:
The authors should explicitly include Step 6 in their methodology and describe:
• Who will be consulted (the "international special interest group" mentioned in the knowledge

translation section should be integrated here)
• How consultation will be conducted (e.g., workshops, interviews, surveys)
• The purpose of consultation (e.g., to validate findings, enhance relevance, inform interpretation)
• How stakeholder input will be integrated into the review findings

- **Response:** Methodology updated to include the JBI framework with the sixth step (consultation with stakeholders) as suggested. **(page 6, line 144 – 163)**

The manuscript incorrectly states that the review will follow PRISMA-ScR (which is correct), but there is inconsistency and potential confusion in the text regarding PRISMA guidelines.

Specific Issues:
• The manuscript correctly references PRISMA-ScR (Tricco et al., 2018) in multiple places
• However, the authors state: "The study selection process will be documented using a PRISMA flow diagram" - this should specifically be a PRISMA-ScR flow diagram

- **Response:** PRISMA guidelines: consistent PRISMA-ScR formatting applied, with corrections to specify the PRISMA-ScR flow diagram and checklist numbering accordingly. **(page 10, line 252)**

Issue: The authors use a Population-Intervention-Outcome-Study design (PIOS) framework without providing a citation or justification. More critically, PIOS is not the most appropriate framework for scoping reviews.
Recommendation: The authors should adopt the PCC (Population, Concept, Context) framework, which is specifically recommended by the JBI Manual for Evidence Synthesis for scoping reviews, OR the PICOS framework for systematic reviews.

- **Response:** The eligibility framework has been modified to base on PCC framework and aligned with our objective **(Page 9, Line 226 – 231)**

Issue: The data extraction plan in Table 2 could be more comprehensive.
Recommendation: Add the following items to the data extraction table:
• Funding sources
• Study setting (e.g., urban, rural, national, community, facility)
• Stakeholders engaged in the intervention
• Theoretical/conceptual frameworks used
• Health system building blocks assessed (WHO framework)

- **Response:** Data extraction table expanded to include study setting, funding source, stakeholders engaged in interventions, theoretical/conceptual framework used, and health system building blocks assessed (WHO framework). ***(Page 10, line 258- 264, 273)***

Issue: The authors state they will use CASP tools for quality appraisal but do not specify which specific CASP checklist(s) they will use (e.g., qualitative studies, cohort studies, etc.). Recommendation: Specify which CASP checklists will be applied based on the study designs identified, and clarify how appraisal results will be presented (e.g., not used to exclude studies but to provide context for interpretation of findings).

- **Response:** Critical appraisal will not be conducted, in line with the primary aim of this scoping review, which is to provide an overview of the included studies. ***(page 11, line 275 – 278; Page 2 line 43)*** this is consistently stated in abstract section and the body.

Issue: The manuscript lacks a dedicated Discussion section, which is an expected component of a protocol paper.
Recommendation: Add a Discussion section that addresses:
1. Anticipated significance and contribution - What will this review add to the existing body of knowledge?
2. Potential limitations beyond those listed in the "Strengths and Limitations" section:
o Restriction to English language publications (may introduce language bias)
o Exclusion of studies published before 2000 (may miss foundational work)
o No formal quality appraisal (may include studies with methodological limitations)
o Potential for publication bias
3. Implications for policy and practice - How will findings inform health system strengthening?
4. Implications for future research - What research gaps might be identified through this review?
5. Anticipated challenges - Challenges in synthesizing diverse evidence (quantitative, qualitative, mixed methods; peer-reviewed vs. grey literature)

- **Response: discussion section added to capture:**
    a. Anticipated significance and contribution. ***(page 12-13, line 317 - 321)***

b. Potential limitations beyond those listed in the "Strengths and Limitations" section. ***(page 13, line 322 - 329)***

c. Implications for policy and practice. ***(page 13, line 330 - 335)***

d. Implications for future research. ***(page 13, line 336 - 340)***

e. Anticipated challenges. ***(Page 13, line 341 - 345)***

Issue: The strengths and limitations section is brief and could be expanded to include methodologically relevant points.
Recommendation: Consider adding:
• Strength: Robust methodological approach based on JBI guidance
• Strength: Comprehensive search across multiple databases and grey literature
• Limitation: Potential for missing non-English publications
• Limitation: Broad scope may limit depth of synthesis for specific components

- . **Response:** Strengths and Limitations expanded to include recommendations: ***(page 2, line 53 - 67)***
  Strength: robust methodological approach based on JBI guidance. ***(page 3, line 57 - 58)***

  Strength: comprehensive search across multiple databases and grey literature. ***(page 3, line 58 - 60)***

  Limitation: potential for missing non-English publications. ***(page 3, line 62 - 63)***

  Limitation: broad scope may limit depth of synthesis for specific components. ***(Page 3, line 65 - 66)***

Issue: The search strategy table (Table 1) is presented in a format that is somewhat difficult to read. Additionally, the authors should clearly state the date when the search will be executed.
Recommendations:
• Format Table 1 more clearly with separate rows for each concept
• State the planned search execution date
• Consider including a sample search strategy for additional databases (at least one other database) in supplementary materials

- **Response:** table 1 formatted **(page 8, line 218),** At the time of submitting this protocol, the search has not yet been conducted; the search strategy will be implemented in September 2026. ***(Page 7, line, 209 – 210)*****.**
